# Fatal Type B Lactic Acidosis Associated With Metastatic Colorectal Cancer: A Case Report With Review of Literature, Pathogenesis, and Treatment

**DOI:** 10.1177/2324709618788101

**Published:** 2018-07-19

**Authors:** Bharatsinh Gharia, Karan Seegobin, Hetavi Mahida, Marwan Shaikh, Trevanne Matthews Hew, Dat Pham

**Affiliations:** 1 University of Florida, Jacksonville, FL, USA; 2 Jersey Shore University Medical Center, Neptune City, NJ, USA

**Keywords:** colorectal cancer, lactic acidosis, literature review

## Abstract

Type B lactic acidosis associated with malignancy is a life-threatening complication and mostly seen in hematological malignancies but can also be seen in solid tumors. We report a rare case of a 64-year-old female diagnosed with metastatic adenocarcinoma of the colon with liver metastasis associated with severe type B lactic acidosis. We discuss pathophysiology, previously reported cases, and their outcomes. The most widely used therapies are bicarbonate infusion, thiamine supplementation, chemotherapy, and supportive care but is associated with poor outcomes, and no standard treatment recommendations are available. Early chemotherapy administration remains the only intervention that has shown some survival benefit. Physicians should be aware and proactive for early diagnosis and management of this condition with further research needed to guide optimal therapy.

## Introduction

Lactic acidosis is a common finding in critically ill patients and is the most common cause of metabolic acidosis in hospitalized patients. There are 3 subtypes of lactic acidosis based on the underlying pathophysiology. Type A lactic acidosis (most common), type B lactic acidosis, and type D lactic acidosis. Type B lactic acidosis is rare and absence of systemic hypoperfusion is the hallmark. It is thought to be caused by toxin-mediated impairment of cellular metabolism but definite etiology remains unclear with multiple hypothesis described in the literature. It can be seen in malignancies, diabetic ketoacidosis, alcohol intoxication, and use of certain medications such as metformin,^1^ propofol, and HIV antiretroviral therapy.^2^ Colon cancer remains the fourth most common cancer with annual incidence of ~135 000 new cases in the United States.^3^ The liver remains the most common site for metastasis for the colorectal cancer. We present a rare case with metastatic colorectal cancer with development of type B lactic acidosis and associated poor outcome. We discuss the possible hypothesis for the pathophysiology of this disorder, outcomes of previously reported cases of solid tumors with type B lactic acidosis, and possible therapeutic interventions to guide clinicians and future research.

## Case Presentation

A 64-year-old Caucasian female presented with complains of right side upper abdominal pain and nausea for 2 months. The pain was progressively getting worse and exacerbated with food. She had lost 10 lbs during this period due to nausea. She did not have any fever, diarrhea, sick contacts, trauma, or recent medication changes. She had chronic hypertension but was not on any medication at home. She had diagnostic colonoscopy 2 months before this admission as outpatient, which showed a partially obstructing mass in the ascending colon, but she was unable to follow-up. She did not have any other surgical history. She was an active smoker with 20 pack-year smoking history. She denied any alcohol or drug use, allergies, and family history of cancer. Her ECOG (Eastern Cooperative Oncology Group) performance status before admission was 1.

She was normotensive (134/76 mm Hg), afebrile, and not tachycardic (94/minute). Her physical examination was remarkable for mild abdominal distention. She had moderate right upper abdominal quadrant tenderness to palpation. There was no guarding, rebound, rigidity, or organomegaly. No masses could be palpated on examination. Her neurological, cardiovascular, pulmonary, and dermatological examination was normal.

Laboratory studies on day of admission showed hemoglobin 8.2 gm/dL (normal = 12.0-16.0 gm/dL), white blood cells 19 200/mL (normal = 4500-11 000/mL), platelets 618 000/mL (normal = 140 000-440 000/mL), serum sodium 131 mEq/L (normal = 135-145 mEq/L), potassium 3.9 mEq/L (normal = 3.3-4.6 mEq/L), chloride 90 mEq/L (normal = 101-110 mEq/L), bicarbonate 14 mEq/L (normal = 21-29 mEq/L), anion gap 27 mEq/L (normal = 4-16 mEq/L), blood urea nitrogen 23 mg/dL (normal = 6-22 mg/dL), creatinine 0.7 mg/dL (normal = 0.6-1.1 mg/dL), and uric acid 5.4 mg/dL (normal = 3-8.2 mg/dL). Her glucose, phosphorus, calcium, urinalysis, and lipase were normal. Lactic acid was elevated 7.2 mmol/L (normal = 0.7-2.7 mmol/L). Liver enzymes were also elevated with aspartate aminotransferase 222 IU/L (normal = 14-33 IU/L), alanine aminotransferase 58 IU/L (normal = 10-40 IU/L), total bilirubin 3.5 mg/dL (normal = 0.2-1.0 mg/dL), normal partial thromboplastin time, and normal international normalized ratio. Arterial blood gas showed pH 7.43 (normal = 7.35-7.45), pCO_2_ 29 mm Hg (normal = 35-45 mm Hg), pO_2_ 52 mm Hg (normal = 80-100 mm Hg), and pHCO_3_ 19.6 mmol/L (normal = 23-29 mmol/L). CEA was 50 ng/mL (normal less than 2.5 ng/mL). HIV and hepatitis tests were negative.

**Figure 1.**
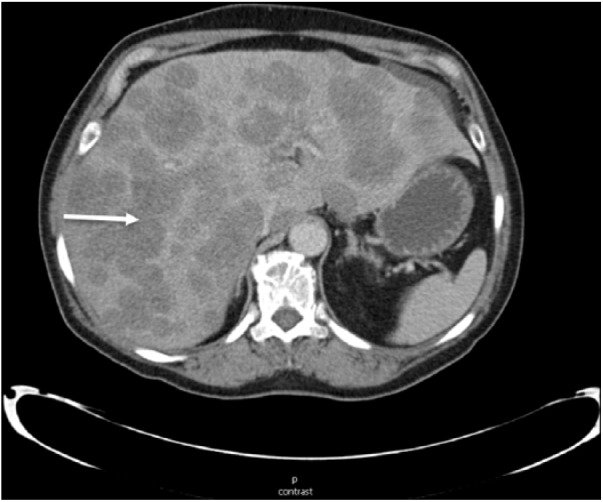
Computed tomography scan of the abdomen suggesting diffuse hypodense liver lesions (arrow) suggestive of metastatic disease.

**Figure 2.**
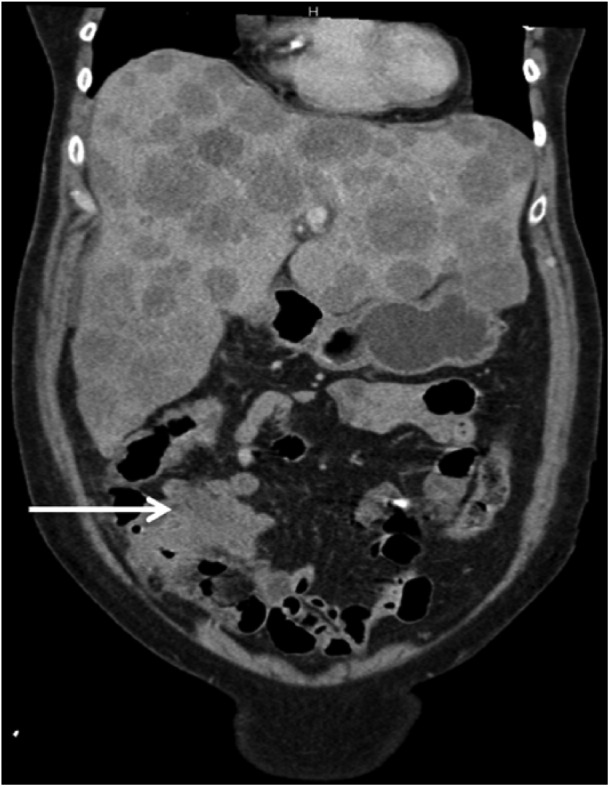
Computed tomography scan of the abdomen (coronal plane) demonstrating primary colon mass (arrow) and diffuse hepatic lesions.

Radiological evaluation with computed tomography scan of the chest, abdomen, and pelvic showed multiple subcentimeter pulmonary nodules, diffuse hypodense lesions throughout the liver resulting in pseudo-nodular appearance of the hepatic contour, subcentimeter retroperitoneal lymph nodes, and within the proximal ascending colon approximately 3.9 cm mass (Figures 1 and 2).

Ultrasonogram of the abdomen showed no intrahepatic or extrahepatic ductal dilatation. The patient was admitted for further evaluation. She was started on broad spectrum antibiotics. Blood, urine, and stool cultures were obtained. There was no evidence of organ hypoperfusion, sepsis, drug use, or malabsorption. The cultures remained negative. After ruling out other causes of lactic acidosis it was deemed due to malignancy associated lactic acidosis. The patient was continued on intravenous fluid with addition of bicarbonate with multivitamin supplementation. On day 3 the patient had liver biopsy of one of the lesions in the liver. Throughout the hospital stay, her laboratory parameters were progressively getting worse, as shown in Table 1.

**Table 1.** Laboratory parameters.

	Hospital Day					
	1	2	3	4	5	6
Serum potassium (normal = 3.3-4.6 mEq/L)	5.2	4.9	4.4	4.3	5.2	5.3
Serum creatinine (normal = 0.6-1.1 mg/dL)	0.7	0.6	0.6	0.5	0.6	0.6
Serum bicarbonate (normal = 21-29 mEq/L)	14	17	19	18	18	15
Blood pH (normal = 7.35-7.45)	7.43	7.43	7.46	7.43	6.99	7.05
Serum lactic acid (normal = 0.7-2.7 mmol/L)	7.2	8.2	10.2	9.4	15.3	20.1

**Figure 3.**
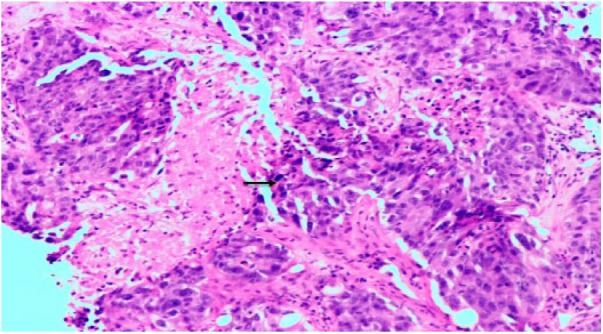
Biopsy of the liver mass demonstrated poorly differentiated adenocarcinoma with marked desmoplasia, sheets of cells with minimal gland formation, and necrosis (arrow) on hematoxylin-eosin staining (magnification 10×).

On day 6 of admission the patient became confused, hypoxic, and unable to maintain airway. Arterial blood gas showed pH 6.99 (normal = 7.35-7.45), pCO_2_ 46 mm Hg (normal = 35-45 mm Hg), pO_2_ 68 mm Hg (normal = 80-100 mm Hg), and pHCO_3_ 11 mmol/L (normal = 23-29 mmol/L). She was intubated for airway protection. Biopsy of the liver mass demonstrated metastatic poorly differentiated adenocarcinoma (Figure 3) with immunohistochemistry stains positive for CK-20, CDX-2, and negative for CK-7, suggesting colorectal primary.

The family was informed about her condition and they decided against intensive care or aggressive interventions and requested comfort measures only with hospice care. The patient passed away on the sixth day of hospitalization.

## Discussion

Lactic acidosis is defined as serum lactate level more than 4.0 mmol/L (normal range = 0.7-2.7 mmol/L). The underlying mechanism for elevated lactate is increased production of lactic acid, decreased excretion, or combination of both. Type A lactic acidosis can be seen in conditions of decreased tissue perfusion including heart failure, sepsis, and cardiopulmonary arrest. Type D lactic acidosis can be seen in patients with gastrointestinal malabsorption and short bowel syndrome, and the etiology is thought to be intestinal bacterial overgrowth with excessive carbohydrate delivery to the small bowel leading to excess D-lactate production and absorption.^4^ Type B lactic acidosis can be associated with malignancy, and the pathophysiology of this condition is unclear and several hypotheses are proposed in the literature.

The liver plays a central role in lactate metabolism as 80% of the lactate is metabolized via gluconeogenesis to glucose in the liver and the remainder by the kidneys, and more than 90% of cases of solid tumors associated with type B lactic acidosis have some degree of liver involvement by the malignancy (Table 2). In contrast, there are multiple cases of hematological malignancies with minimal or no involvement of the liver with development of this complication.^5^ The liver remains the most common site for metastasis for colorectal carcinoma with approximately 20% newly diagnosed patients presenting with metastatic disease.^6^ In spite of having large number of patients with metastasis to the liver from solid tumors, the incidence of this complication remain low, suggesting multifactorial plausibility of this complication.

**Table 2.** Reported cases.

Reference	Report Year	Diagnosis	Age	Sex	pH	Lactate (mmol/L)	Bicarbonate (mEq/L)	Liver Mets	Transaminitis	Treatment	Outcome
7	1998	Metastatic small cell	70	Male	7.29	15	10.6	No	No	Bicarbonate therapy, chemotherapy	Acceptable (5 months)
8	2000	Cholangiocarcinoma	70	Male	7.11	12.5	12.2	Yes	Yes	Dialysis	Poor (6 days)
9	2002	Undifferentiated carcinoma	25	Female	7.08	19	10.2	Yes	Yes	Bicarbonate therapy, dialysis	Poor (days)
10	2004	Undifferentiated carcinoma	14	Female	—	22	—	—	—	Chemotherapy	Acceptable (2 months)
11	2006	Metastatic small cell	64	Male	7.18	15.8	9	Yes	Yes	Bicarbonate therapy, dialysis	Poor (days)
12	2011	Metastatic colon	44	Female	7.2	11	6	Yes	Yes	Bicarbonate, chemotherapy	Acceptable (months)
13	2011	Metastatic prostate	71	Male	7.07	22	7.2	Yes	Yes	Bicarbonate therapy, chemotherapy	Poor (days)
14	2011	Metastatic breast	86	Female	7.35	7.5	14	Yes	Yes	Bicarbonate therapy	Poor (days)
15	2012	Metastatic pancreatic	56	Female	7.1	—	—	Yes	Yes	Supportive	Poor (days)
16	2014	Undifferentiated carcinoma	76	Female	7.27	11.9	9	Yes	Yes	Bicarbonate therapy, dialysis	Poor (days)
17	2015	Metastatic gastric	81	Female	7.35	6.1	16	Yes	Yes	Fluids	Poor (days)
18	2017	Metastatic small cell	73	Male	6.8	24	11	Yes	Yes	Bicarbonate therapy	Poor (days)
Our patient	2017	Metastatic colon	64	Female	6.99	19	11	Yes	Yes	Bicarbonate therapy	Poor (days)

The Warburg effect has been described in which the tumor cells switch their metabolism to lactic acid pathway leading to increased intracellular NADPH, redirection of glucose to pentose phosphate shunt, and increased nucleoside and amino acid biosynthesis, which are used by the tumor cells to replicate^16^ even in hypoxic tumor environment.

Thiamin is a cofactor for the enzyme pyruvate dehydrogenase, which is necessary for conversion of pyruvate to acetyl CoA, which then enters the Krebs cycle. In thiamin deficiency, the excess pyruvate is converted to lactate by the hormone lactate dehydrogenase leading to lactic acidosis. This can be seen in patients on total parenteral nutrition. Supplementation of thiamin in this condition can improve lactic acidosis^19^

Tumor necrosis factor-α is an inflammatory cytokine released by the hematological malignancies and is thought to inhibit pyruvate dehydrogenase and increase lactate production, but its role in solid tumor is not clear, although some tumors overexpress hexokinase- and insulin-like growth factor, which can increase glycolysis and pyruvate production.^20^

We believe tumor vascularity has an important role in this condition, as tumors with very high proliferation index outgrow their vascular supply, overexpression of glycolytic enzymes drives increased glycolysis and anaerobic metabolism in relatively hypoxic tumor microenvironment, and surrounding normal tissues develop ischemia and necrosis. Production of lactate exceeds the capacity of hepatic clearance in presence of diffuse liver metastasis, leading to severe metabolic acidosis.

Most reported cases of type B lactic acidosis are associated with hematological malignancies. Multiple case reports have been reported with patients of multiple myeloma,^21^ lymphoma, and leukemia^22^ complicated by type B lactic acidosis. The data on solid tumors are limited, and we performed literature search using PubMed with the search terms “type B lactic acidosis” and “lactic acidosis and colon cancer.” Our case represents the second case in the literature in the last 20 years, associating colorectal cancer with severe type B lactic acidosis. Table 2 outlines solid malignancies associated with type B lactic acidosis reported from 1997 to 2017 featuring laboratory finding and outcomes.

Analysis of the above-mentioned cases suggest metastatic small cell carcinoma (23%) and undifferentiated carcinoma (23%) remain the leading causes of this complication. Mean age of diagnosis is 61 years, females are affected more than males (61.5% vs 38.5%), mean pH was 7.14, and mean lactic acid level was 15.8 mmol/L. More than 90% of the patients with this condition had some degree of liver involvement with cancer, and the most patients were treated with bicarbonate therapy. Overall, the prognosis appears poor with 77% mortality rate in few days to weeks. The small percentage of patients (23% from above) who were able to receive chemotherapy had relatively better survival (months) compared with the remainder. It remains unclear if better baseline performance status and laboratory parameters make these patients eligible to tolerate chemotherapy and hence the acceptable prognosis. It is also of prime importance that physicians take into consideration the toxicities of the chemotherapy. Multiple chemotherapy regimens are approved for first-line metastatic colon cancer,^23^ such as 5-flourauracil, oxaliplatin, irinotecan, capecitabine (with or without combination with VEGF inhibitor or EGFR inhibitor) and most of them are either contraindicated or need dose adjustment in cases of severe hepatic dysfunction, which is present in most of these patients.

Management of this condition is controversial and no standard therapy recommendations are available. Bicarbonate therapy has been used in most patients (Table 2) but appears ineffective. Thiamin supplementation is advised as it has shown benefit^5^ with minimal toxicity risk. Due to high mortality rate associated with this condition, early intensive care unit monitoring should be considered. If deemed appropriate and pathology is available then an optimal chemotherapy regimen based on the primary malignancy should be considered as it has shown survival benefit than supportive care alone. All the reported cases are of untreated or newly diagnosed patients and none had any prior therapy for the metastatic cancer. This suggests delay in chemotherapy administration in new patients with metastatic cancer, especially with extensive hepatic involvement this should be avoided to prevent complications. Multidisciplinary care involving intensivist, nephrologist, and oncologist should be advocated to manage this condition.

## Conclusion

This was a rare case of severe type B lactic acidosis associated with metastatic adenocarcinoma of the colon. All patients with metastatic cancer with extensive hepatic involvement and unexplained high anion gap metabolic acidosis with elevated serum lactate in the absence of other causes of lactic acidosis should be considered to have this fatal complication. Currently there is no standard of care therapy available. Early administration of chemotherapy if feasible should be considered. Prompt recognition, randomized trials using novel pharmacotherapy agents to develop optimal management paradigm, and increased awareness of this complication among physicians is crucial to conquer this underrecognized metabolic oncologic emergency.
